# Study on the differences and influencing factors of spatial distribution of population aging at township scale: a case study of township research units in Anshun City, China

**DOI:** 10.3389/fpubh.2024.1351395

**Published:** 2024-03-28

**Authors:** Xuebin Zhang, Jing Shi, Meng Chao, Junfeng Yin

**Affiliations:** ^1^College of Geography and Environment Science, Northwest Normal University, Lanzhou, China; ^2^Key Laboratory of Resource Environment and Sustainable Development of Oasis, Lanzhou, China; ^3^Ecological Environment Emergency and Monitoring Center, Zhengzhou, China; ^4^State Key Laboratory of Earth Surface Processes and Resource Ecology, Beijing Normal University, Beijing, China; ^5^Center for GeoData and Analysis, Faculty of Geographical Science, Beijing Normal University, Beijing, China

**Keywords:** population aging, spatial difference, influence factors, multi-scale geographical weighted regression, Anshun City, China

## Abstract

An aging population is one of the main features of China's current population structure, and it is a key area that needs attention to achieve high-quality population development. Because of its unique geographical environment, economic conditions, and sociocultural background, the study of population aging in the karst region of southwest China is particularly important. However, there is a lack of research exploring the regional differentiation of population aging and its influencing factors in the karst regions of southwest China. In light of this, we chose Anshun City, located in Guizhou Province's southwest area, as the case study area. We used the Lorenz curve and spatial autocorrelation to study the differences in the spatial distribution pattern of population aging and introduced multi-scale geographical weighted regression to explore its influencing factors. The results show that Anshun City's older people population proportion (OPP) is generally high with more than 7% of the older people there, making it part of an aging society. The OPP appeared high in the east and low in the west in spatial distribution; the older people population density (OPD) revealed a gradually increasing trend from south to north. At the township scale, both the OPP and the OPD showed significant spatial positive correlation, and the spatial agglomeration characteristics were obvious. OPD and OPP have a positive spatial correlation at the global level, and townships with similar OPP or OPD were spatially adjacent. The spatial distribution characteristics of population aging are the consequence of complex contributions such as natural, social, economic, and karst factors. Further, the spatial distribution pattern of aging is determined by a variety of influencing factors, which have different directions and intensities. Therefore, it is necessary to formulate and implement corresponding policies and strategies to deal with the aging problem in the future.

## 1 Introduction

Population aging refers to the dynamic process of an increasing proportion of older adults in the population (1). Along with the expansion in scale and acceleration of population aging globally, population aging has become an important issue that needs to be addressed urgently by all, especially in developing countries (2) where its impact is far-reaching and diverse. China, as the largest developing country in the world, has attracted wide attention in recent years for the trend, speed, and characteristics of population aging, along with new characteristics such as “rapid growth trends, obvious regional differences, and diverse provincial differences” (3). According to China's seventh national census in 2020, the proportion of people aged 65 and above had reached 13.5%, while the proportion of those aged 60 and above had reached 18.7%. The intensification of population aging has had a profound impact on China's society, economy, medical and health care, family, and other aspects (4). Socially, the increase in the older adult population intensifies the social burden and places higher requirements on the social security system. Economically, aging may lead to labor shortages, labor market imbalances, and other problems, which will have a negative impact on China's economic development. In terms of medical and health care, with an increase in the older adult population, the demand for medical resources is also increasing, which places higher requirements on the medical and health system. In terms of family, aging may lead to changes in family structure, such as the increase in the phenomenon of empty nesters, which brings more challenges and responsibilities to families. Therefore, it is necessary to study the spatial differences and factors that influence population aging to help the government formulate population development policies and promote the coordination of population, resources, and environment (5).

As one of the popular topics in population geography, population aging has been explored and studied by many scholars. At present, the relevant research on population aging focuses on the following points. First, it focuses on the measurement and calculation of population aging indicators. In general, single indicators such as older people population proportion (OPP), older people population density (OPD), and older people dependency ratio (ODR) were used to measure the level of population aging (6). However, some scholars have proposed that composite indicators can more precisely reflect the severity of population aging. For example, Xu et al. (7) established a comprehensive population aging index system composed of the number, proportion, and density of the older adult population.

Secondly, the study of population aging focuses on the behavior analysis of the time evolution characteristics and spatial differentiation characteristics of population aging. The analysis of temporal evolution is based chiefly on census data, describing the variation trend and evolution stage of the long-term series of older people at the national or provincial level (8). For example, Zhou et al. (9) pointed out that the overall trend of the older adult population proportion has been fluctuating increasingly from 1990 to 2016 in China, and all provinces except Tibet have entered an aging society. Kashnitsky et al. (10) found that the differences in the age structure of the population across urban and rural regions in the European NUTS-2 region had no significant difference over time. Spatial differentiation usually entails focusing on a specific region of a medium scale as the research object, and through spatial autocorrelation analysis (11), standard deviational ellipse (12), and kernel density estimation (13), the agglomeration types and spatial correlation of population aging are studied. For instance, Guan et al. (14) measured the level of population aging in 100 districts and counties in Liaoning Province from 1990 to 2010 and found that the spatial distribution of population aging in the province showed an unceasingly intensifying northeast-southwest pattern. County areas along the Shenyang-Dalian line showed a stronger effect of aging within the whole province. Similarly, Zhao et al. (15) pointed out that the Chinese population aging showed noticeable regional differences, and the spatial distribution of the older adult population tended to be concentrated or dispersed based on different cities. Li et al. (16) reported that the population aging in the Shandong Province at the township scale had a positive spatial correlation in the whole area, and different townships showed apparent spatial differences. However, small-scale studies have accurately described the different characteristics of population aging (17) and also identified new problems, such as regional differences in population aging that are amplified at the micro-scale.

Thirdly, focusing on the study of the factors affecting the spatial differentiation of population aging helps in the discussion of countermeasures. These factors mainly include natural factors, social factors, and economic factors. The manner in which physical geographic factors influence the spatial differentiation of regional population aging is a complex and important problem and includes natural factors such as temperature, rainfall, elevation, slope, and so on (18). Some researchers have proposed that unique environmental factors in specific regions significantly impact spatial differentiation of population aging. For example, Ying et al. (19) studied the impact of rocky desertification on the aging population in Guizhou Province and found that an improvement in the rocky desertification has a stronger effect on population aging. Social factors mainly include migration rate, medical facilities level, education level, and ethnic structure (20, 21). Economic factors include GDP per capita, urban and rural structure (URS), land use, etc. (5, 22).

Another important issue of population aging research is to explore whether the mechanism of different scale factors is unified. For example, Wu et al. (23) found that the impact of migration rate on population aging at the provincial level or larger scale was positive, while Zhao et al. (15) reported that migration rate was negatively related to population aging at the county level.

In terms of research methods, the Bayesian Space-Time model (24), geo-detector (22), quantile regression model (25), and geographically weighted regression (GWR) (26) are the main methods used in population aging-related research. With regard to research areas, current population aging studies tend to choose developed cities for case studies leading to subjective and one-sided research results that cannot accurately reveal the overall characteristics of population aging (27).

The karst mountain area is a unique natural landscape in southwest China. Its topography, geomorphology, and geological features are unique and rich in diversity. Its special geological features and the fragile ecological environment in the face of heightened human activities have resulted in the karst area of southwest China becoming significantly vulnerable. The fragile ecological environment and strong human activities in southwest China's karst area make it highly vulnerable. This vulnerability is not only reflected in the deterioration of the natural environment but also in the lagging economic and social development. This has further triggered the outflow of a large number of the local young population (28), resulting in the imbalance of the local population structure and the increasingly serious problem of population aging.

In general, population research is easily affected by research scale, and the smaller the scale of the basic research unit, the more helpful it is to scientifically reveal the spatial behavior and interactive characteristics of the temporal behavior of population structure development. Therefore, in this study, we chose to examine the differences in spatial distribution and the influencing factors of population aging in typical cities in the karst landform area based on research at the level of towns and villages. However, it should be pointed out that a more detailed study at the township scale also means limited access to various basic population data compounded by relatively significant collection and collation difficulties, resulting in differences in the degree of direct reflection of the selected indicators on the spatial distribution of population aging. Consequently, township units in Anshun, a typical city in southwest China, were selected as the case study area. Lorenz curve and spatial autocorrelation were used to study the differences in the spatial distribution pattern of population aging, and multi-scale geographical weighted regression (MGWR) was introduced to explore the influencing factors. The result will provide a decision-making basis for solving the problem of population aging in the karst mountains.

## 2 Materials and methods

### 2.1 Study area

**Figure 1:**
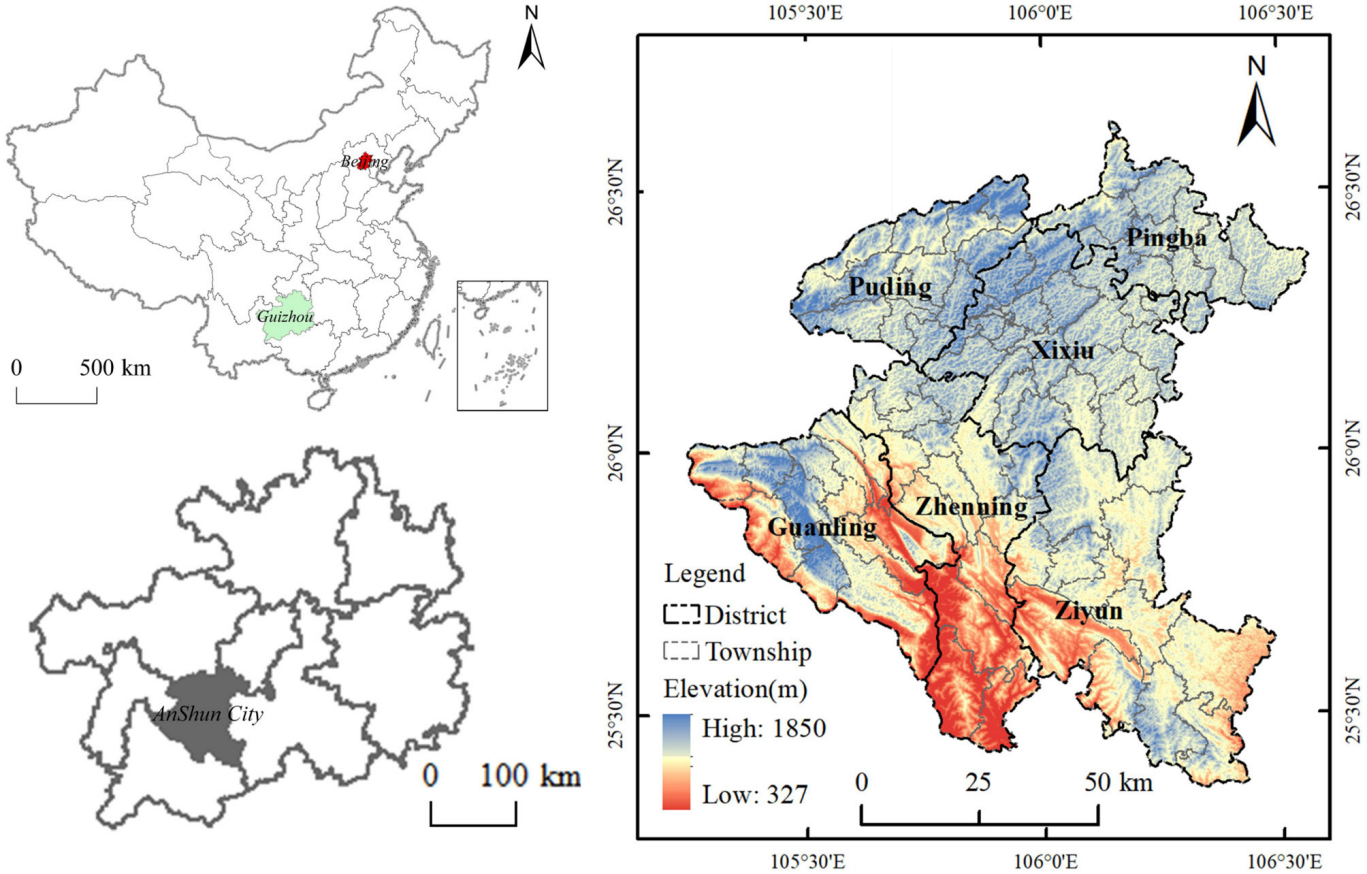
Study area.

Anshun City, with an area of 9,267 sq km, is located in the central and western part of Guizhou Province (Figure 1), on the eastern slope of the Yunnan-Guizhou Plateau, which is a typical karst landform area. The region is elevated high in the northwest and low in the southeast, with many mountains and rivers crisscrossing. The lowest elevation is 327 m and the highest elevation is 1,850 m. According to the seventh national census bulletin of Anshun City in 2020, the city has a permanent population of 2,470,630 people. Among them, the older adult population aged 60 and above is 392,951 people, accounting for 15.90% of the total population. The older adult population aged 65 and above is 286,962, accounting for 11.61% of the total population. Compared with 2010, the proportion of people aged 65 and above has increased by 2.93 percent. In the six counties and districts under the jurisdiction of Anshun City, the proportion of the older adult population aged 60 and above exceeds 15%, and the proportion of the older adult population aged 65 and above exceeds 10%. Among them, Zhenning County has the highest proportion of the population aged 60 and above, reaching 16.54%. The total population of Anshun City is moderate, but the population distribution is uneven, mainly concentrated in the central city and the area along the roads. With the acceleration of the urbanization process and an increase in population flow, the population structure of Anshun is also changing. The aging problem is gradually getting prominent, and the city is faced with human resource problems such as brain drain and labor shortage. In China, the rural-urban structure is unique, with subdistricts often referred to as “streets.” The streets (subdistricts) of Xinan, Xuxiu, Ningxi, Baihe, and Yunling were divided from the pre-existing streets in 2019 and lack a clear boundary in the most recent Anshun map. We integrated these streets with the streets they were originally a part of in order to guarantee the reliability and correctness of the statistics data. Finally, 87 township research units were selected as research cases.

### 2.2 Data source

The population aged 65 and older was selected as the research object in this study. We obtained the township boundaries of Anshun from the Guizhou Provincial Platform for Common GeoSpatial Information System (https://guizhou.tianditu.gov.cn/). OPP, OPD, migration rate, and education level were collected from the Seventh *National Census Report* of Anshun. Digital elevation model (DEM) and Landsat8 image were downloaded from Geospatial Data Cloud (http://www.gscloud.cn/sources/). Temperature, rainfall, GDP, and karst landform data were obtained from a 1 km raster data set provided by the Resource and Environmental Science and Data Center (http://www.resdc.cn/). PM2.5 data were collected from a (0.01° × 0.01°) data set provided by the Atmospheric Composition Analysis Group, Washington University (https://sites.wustl.edu/acag/datasets/surface-pm2-5/). URS and ethnic structure were obtained from the *Anshun Statistical Yearbook 2020* (http://www.anshun.gov.cn/zfsj/tjnj/). Medical data were crawled from Gaode map. Karst proportion and karst lithology were obtained from 1:1,000,000 Digital Geological maps of the People's Republic of China (29). River data was collected from 1:250,000 vector map data provided by the National Catalog Service for Geographic Information (https://www.webmap.cn/main.do?method=index/). Construction land and cultivated land were downloaded from a 10 m raster data set provided by World Cover (https://viewer.esa-worldcover.org/worldcover/).

### 2.3 Research method

#### 2.3.1 Lorenz curve

The Lorenz curve (also known as the frequency accumulation curve) is a tool used to analyze the distribution of a regional population. The shape of the Lorenz curve can reflect the uniformity of population distribution. If the curve is close to a diagonal, it means that the population is relatively evenly distributed across regions. The further the curve deviates from the diagonal, the more uneven the population distribution, that is, the population density in some areas is much higher than in others (30). We also calculated the Gini coefficient based on the Lorenz curve; its value ranges between [0,1], where 0 represents the absolute equilibrium of population distribution, and 1 represents the complete imbalance of population distribution (31).

#### 2.3.2 Spatial autocorrelation

Spatial autocorrelation reveals the spatial aggregation degree of a particular geographical object's attribute value and its adjacent units' attribute value (14). It is divided into global spatial autocorrelation and local spatial autocorrelation.

##### 2.3.2.1 Global spatial autocorrelation

The Moran's I index was used to analyze the global spatial autocorrelation of population aging at the township scale in Anshun City. The global correlation of population aging at the township scale can be calculated as follows:


(1) $$\begin{array}{l} {\text{Moran}}^{\prime }\text{s}I=\frac{\overset{n}{\underset{i=1}{\sum }}\overset{n}{\underset{j=1}{\sum }}W_{ij}\left(x_{i}-\bar{x}\right)\left(x_{j}-\bar{x}\right)}{S^{2}\overset{n}{\underset{i=1}{\sum }}\overset{n}{\underset{j=1}{\sum }}W_{ij}} \end{array}$$

Where, Moran's *I* represents the global autocorrelation coefficient, *n* represents the number of research units, *W*_*ij*_ is the spatial weight, x is the average, and *x*_*i*_ and *x*_*j*_ represent the observed values of administrative units *i* and *j*, respectively. Moran's I index ranges between 0 and 1, Moran's I > 0 indicates a global positive correlation, with similar values clustered spatially Moran's I > 0 indicates a positive spatial correlation, Moran's I < 0 indicates a global negative correlation, and the values of neighboring towns differ greatly, indicating that the global distribution is dispersed. In theory, when Moran's I = 0 indicates no correlation or random distribution (32).

##### 2.3.2.2 Local spatial autocorrelation

Local spatial autocorrelation reflects the degree of spatial correlation between the attribute value of a spatial object and the attribute value of its neighboring region and is used to explore the clustering degree or heterogeneity of the attribute value of a spatial object in the local space. It can be used to represent the local agglomeration of population aging distribution at the township scale. The local spatial autocorrelation measure can be represented by LISA. The calculation formula is shown in Equation 2:


(2) $$\begin{array}{l} \text{LISA}=\frac{\left(x_{i}-\bar{x}\right)}{S^{2}}\overset{n}{\underset{j=1}{\sum }}W_{ij}\left(x_{j}-\bar{x}\right) \end{array}$$

Where, *S*^2^ represents the variance, other variables are consistent with those described in publicity (Equation 1). When the LISA value is positive, it indicates high-high concentration (H-H type) or low-low concentration (L-L type), That is, the aging degree of the population in this town and the surrounding towns is both high or low. When the LISA value is negative, it means high-low agglomeration (H-L type) or low-high agglomeration (L-H type), that is, the population aging degree of the township is inconsistent with the trend of population aging degree of the surrounding townships.

#### 2.3.3 Multiscale geographically weighted regression model

In the past, researchers have adopted various statistical and analytical methods, such as multiple linear regression (MLR), spatial regression (SR) geographical weighted regression (GWR), and so on. However, in the MLR it is easy to ignore the spatial interaction of influencing factors, while in the SR it is easy to ignore the spatial non-stationarity of influencing factors. Although the GWR can effectively detect spatial non-stationarity, it ignores the consideration of the differential scale effect of influencing factors, which may lead to errors in the regression results (33, 34). Multiscale geographical weighted regression (MGWR) is an extension and supplement to the traditional GWR, which can provide different spatial smoothing levels and specific bandwidths for each variable, generate a spatial process closer to the actual effect (35), and carry out regression fitting analysis on different spatial scales. Thus, the spatial heterogeneity of each variable's influence on the dependent variable at different spatial scales can be explained more accurately, the above method problems can be effectively solved, and the scale differences of different influencing factors can be identified (36). In this study, MGWR was used to explore the influencing mechanism and spatial heterogeneity of population aging spatial differences and compare the differences of each influencing factor. The calculation formula is shown in Equation 3:


(3) $$\begin{array}{l} y_{i}=\beta_{0}\left(u_{i},v_{i}\right)+\overset{n}{\underset{j=1}{\sum }}\beta_{bwj}\left(u_{i},v_{i}\right)x_{ij}+\varepsilon_{i} \end{array}$$

Where, *y*_*ij*_ represents the explained variable, *x*_*ij*_ is the explanatory variable, β_0_(*u*_*i*_*, v*_*i*_) represents the location intercept of research unit *i*, β_*bwj*_ is the local regression coefficient of the *j*th explanatory variable of research unit *i*, and *bwj* represents the bandwidth used when estimating the *j*th explanatory variable, ε_*i*_ is the error term.

## 3 Results and analyses

### 3.1 Spatial distribution characteristics of population aging

To specifically illustrate the spatial distribution characteristics of population aging in Anshun City, we referred to the classification of population aging proposed in previous studies (5, 37), then divided the OPD and OPP into four and six types, respectively. OPD ≤ 25 person/km^2^ was the scarce-density type, 25 person/km^2^ < OPD ≤ 50 person/km^2^ was the low-density type, 50 person/km^2^ < OPD ≤ 100 person/km^2^ was the medium-density type, and OPD 100 person/km^2^ was the high-density type. OPP < 4% was young-proportion type, 4% OPP 5.5% was adult-proportion type, 5.5% ≤ OPP 7% was adult -proportion type, 7% OPP < 10% was old-proportion type, 10% ≤ OPP < 14% was old -proportion type, and OPP ≥ 14% was old -proportion type.

**Figure 2:**
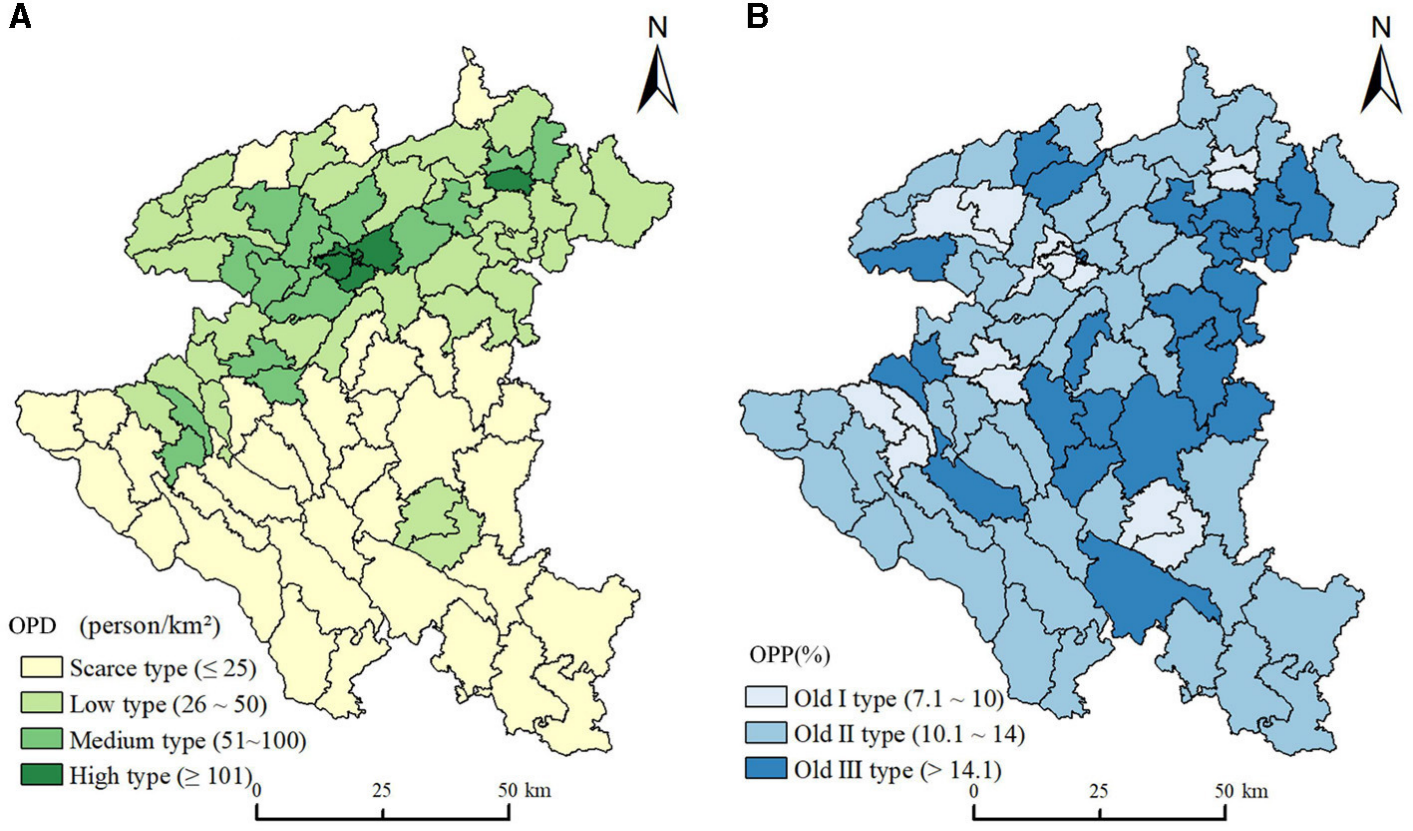
Spatial distribution of the older people population. **(A)** Older people population density. **(B)** Older people population proportion.

As shown in Figure 2A, the density type in the townships of Anshun was dominated by the scarce-density type. Specifically, approximately 35 townships showed the scarce-density type and accounted for 40.23%, indicating that the OPD of Anshun City was generally low. Figure 2B shows that OPP in all townships exceeded 7%, which means Anshun has entered the aging society overall. Approximately 48 townships had old-proportion type accounting for 55.17%. The OPD showed a trend of increasing gradually from south to north in space. Most of the towns in the south of Anshun belong to the scarce-density type, but Wufeng Street (34 person/km^2^) and Songshan Street (35 person/km^2^) appeared to be of low-density type. The density type of the northern townships was primarily low-density or medium-density, occasionally interspersed with high-density type represented by Dongguan Street (293 person/km^2^), Xihang Street (417 person/km^2^), and Huaxi Street (209 person/km^2^). OPP showed a trend of increasing gradually from west to east and appeared “high in the east and low in the west” in spatial distribution. The townships with higher OPP were mainly distributed in the east of Anshun City, showing a thin distribution. Among all the townships in the city, the OPP of Huangla town was the highest (19.61%), indicating that the town was vulnerable to the serious impact of population aging. On the contrary, the western township mainly presented old -proportion type and also contained a few old -proportion type such as Dingyun Street (8.3%) and Chuandong Street (7.7%).

**Figure 3:**
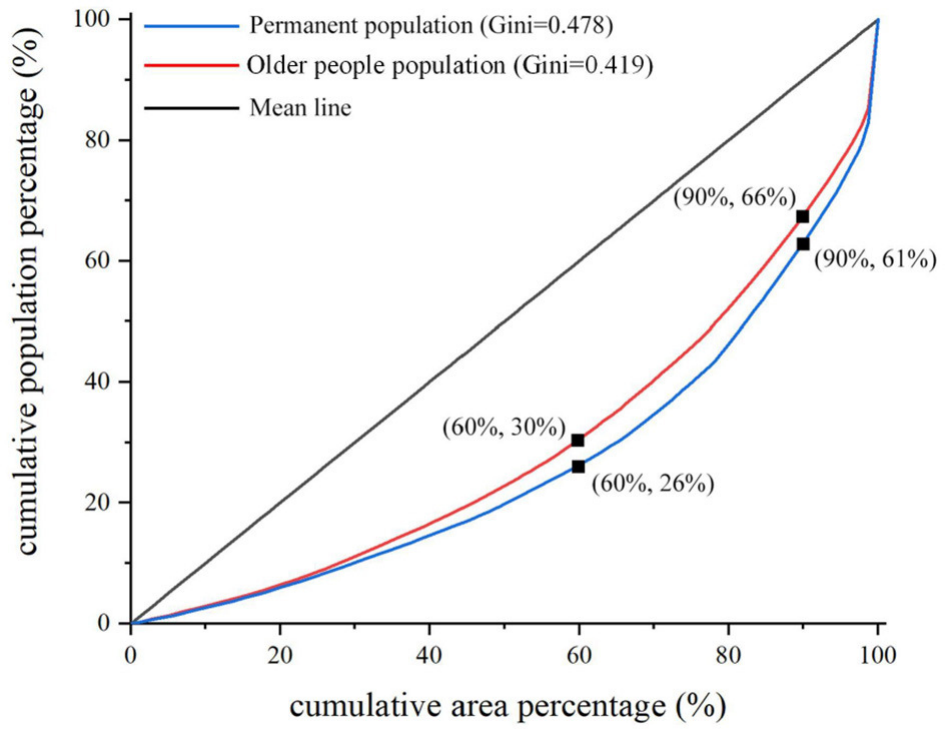
Lorenz curve.

The Lorenz curve for the older people population was plotted by sorting the OPD of each township in ascending order, calculating the cumulative area percentage and the cumulative population percentage, and then calculating the Gini coefficient. The permanent population Lorenz curve was also introduced for comparison. As shown in Figure 3, although the curvature of the population of older people Lorenz curve was smaller than the permanent population Lorenz curve, it still presented a substantial deviation from the mean line. Meanwhile, the Gini coefficient of the older people population (0.419) was also smaller than that of the permanent population (0.478), and greater than the absolute average value. This indicated that although the spatial difference between the population of older people was less than that of the permanent population, it still showed significant unbalanced characteristics in spatial distribution. Specifically, 26% of the permanent and 30% of the older people population were distributed on 60% of the land area of Anshun. When the cumulative land area increased to 90%, the proportion of the permanent and older people populations only increased to 61% and 66%. However, 39% of the permanent and 34% of the older people population gathered on narrow land with only 10% of the area (Figure 3). The distribution of the older people population in the vast majority of townships was relatively scattered. Still, a few townships were oversaturated with the older people population, showing the characteristics of “small concentration and large scattering.”

### 3.2 Spatial correlation characteristics of population aging

The global Moran's I index of OPD and OPP were calculated by GeoDa (Table 1). Table 1 shows that Moran's I index of OPD = 0.487, *p* < 0.01, *Z* score > 2.58 (99% confidence interval), while Moran's I index of OPP = 0.186, *p* < 0.01, *Z* score > 2.58 (99% confidence interval). These results indicated that OPD and OPP have a positive spatial correlation in global scope, and townships with similar OPP or OPD were spatially adjacent. We then drew LISA cluster maps to explore the spatial association of OPP and OPD in local scope, respectively.

**Table 1:** Global autocorrelation analysis.

**Dependent variable**	**Moran's I index**	***p*-value**	***Z* score**	**Confidence interval**
OPD	0.487	0.001	8.753	99%
OPP	0.186	0.005	2.974	99%

**Figure 4:**
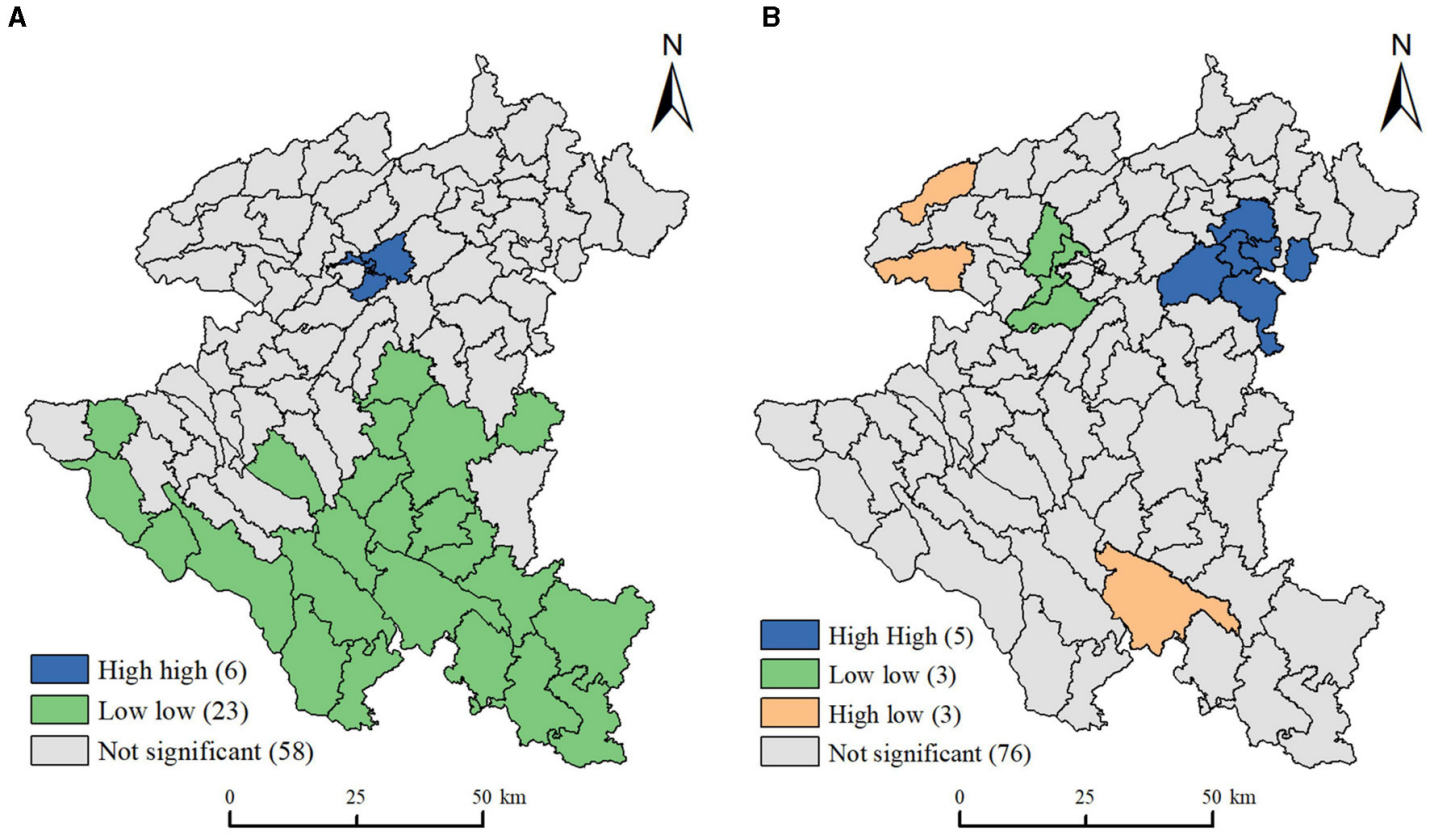
LISA clustering map of population aging index. **(A)** Older people population proportion. **(B)** Older people population density.

From Figure 4A, we can see that the OPD in local scope included high-high and low-low agglomeration types besides not significant units. High-high types of townships were mainly distributed in the central parts of the Xixiu District, which showed that the local spatial population aging index had obvious spatial agglomeration and similarity. The main reason is favorable climate, flat terrain, fertile soils, etc. (31), as well as the relatively high level of economic development, good social welfare conditions, and a perfect medical security system. These factors attracted more older adults to move in, and the density of the older adult population in the region has increased, forming a concentration of older adult population. Low-low types of townships were distributed in the southern part of Anshun in the form of clusters. The significant characteristics of these townships are relatively poor natural environmental conditions (complex terrain, poor soil, fragile ecology), relatively backward social and economic development, and poor social welfare benefits and an imperfect medical security system for the older adult population, leading to their outflow, thus forming a low concentration area for OPD development.

As shown in Figure 4B, OPP in local scope included high-high, low-low, and high-low agglomeration types besides not significant units. High-high types of townships were concentrated in the northeast boundary of Anshun, which has relatively flat terrain and convenient transportation, the region was geographically close to Guiyang, the capital of Guizhou Province. Further, economic reasons guide young people to migrate out of the city (38), resulting in the region having a larger share of older adults, which worsens population aging in this area. Low-low types of townships were concentrated in Songqi town, Yaopu town, and Baiyan town, showing a coupling relationship with townships with high economic levels. The main reason is that towns with higher economic development levels attract a large number of young migrant workers, thus diluting the proportion of the older adult population in the region. High-low types of townships were mainly located on the western and southern borders of Anshun City. These types of townships border on streets under the jurisdiction of the county, such as Wufeng and Chuandong streets, which are significantly affected by the population siphon effect (14); these areas displayed a higher level of population aging than surrounding areas.

### 3.3 Influencing factors of spatial distribution differences of population aging

#### 3.3.1 Selection of influencing factors

The spatial distribution characteristic of population aging at the township scale was influenced by complex and comprehensive factors. We referred to previous empirical research to identify the influencing factors (9, 39, 40) and the unique geographical characteristic factors of the karst mountainous areas (19, 31). Based on the actual regional development, we selected 18 variables from four dimensions including natural factors, social factors, economic factors, and karst factors. The specific indicators are listed in Table 2.

**Table 2:** Selection of influencing factors.

**Dimensions**	**Indicators**	**Calculation and processing**
Natural factors	Elevation	Extracted by 30 m DEM data
	Slope	Extracted by 30 m DEM data
	Temperature	Extracted from the 1 km annual average temperature raster dataset
	Rainfall	Extracted from the 1 km annual average rainfall raster dataset
	River density	Calculated river length/land area
	Air quality	Extracted PM2.5 concentration mean value of PM2.5
Economic factors	GDP per capita	Extracted by China GDP spatial distribution 1 km grid raster dataset
	CLC	Calculated construction land area/land area
	CLA	Calculated arable land area/land area
	URS	Street = 3, urban township = 2, rural township = 1
Social factors	Ethnic structure	Ethnic township = 1, street or non-ethnic township = 2
	Medical level	Calculated the number of hospital POI/permanent population
	Education level	Calculated average years of schooling for the population aged 15 years and older people
	Migration rate	(permanent population-household population)/household population
Karst factors	Karst proportion	Classified karst and non-karst areas based on lithology and extracted the percentage of karst area
	KLI	Geological structures showed vertical distribution, based on the upper, top, and latest stratigraphic age to identify the lithology. The dolomite (with the largest area share of 59.81%) was chosen to represent the lithology; it contained single dolomite, dolomite with impurities, and dolomite and other combinations.
	KLA	Landform types cannot be directly quantified. So, we calculated the area share of each landform type. The low-relief mountain (with the largest area share of 59.21%) was chosen to represent the landforms; it contained low altitude, middle altitude, high altitude, and highest altitude mountains.
	Rocky desertification	Excluded non-rock desertification land types like water and construction land, then extracted rock desertification area based on fractional vegetation cover (FVC) and normal difference rock index (NDRI) in landsat8 image

CLC, Coefficient of Land for Construction; CLA, Coefficient of Land for Arable; URS, Urban and Rural Structure; KLI, Karst Lithology; KLA, Karst Landform.

#### 3.3.2 Analysis of MGWR regression coefficient results

The Pearson correlation coefficient and statistical significance of the different factors with OPP and OPD were assessed through SPSS. As shown in Table 3, the four variables of GDP per capita, migration rate, urban and rural structure, and education level, all passed the 1% or 5% significance test. The elevation variable failed to pass the significance test. The remaining 12 variables passed only a single significance test with OPP or OPD. In this study, the mean strength of the two dependent variables was separated into the following four categories based on the average absolute value of the impact factors: both influence, only influence OPD, only influence OPP, and no influence.

**Table 3:** Correlation analysis with OPD and OPP.

**Indicators**	**OPD**	**OPP**	**Indicators**	**OPD**	**OPP**
Elevation	0.201	−0.037	Medical level	0.116	−0.474^**^
Slope	−0.355^**^	−0.053	Migrate rate	0.430^**^	−0.623^**^
Rainfall	0.221^*^	0.074	URS	0.330^**^	−0.556^**^
Temperature	−0.233^*^	−0.095	Ethnic structure	0.273^*^	−0.123
Air quality	0.481^**^	−0.130	Education level	0.527^**^	−0.361^**^
River density	0.312^**^	0.142	KLA	−0.300^**^	0.015
GDP per capita	−0.273^*^	0.478^**^	Rocky desertification	0.614^**^	−0.025
CLC	0.914^**^	−0.015	Karst proportion	0.213^*^	−0.138
CLA	−0.283^**^	0.144	KLI	0.251^*^	−0.15

^*^Indicates the correlation is significant at the 0.05 level; ^**^Indicates the correlation is significant at the 0.01 level.

The perception of factors influencing population aging at the township scale was not yet unified, which may lead to greater subjective errors if we choose only a few indicators. To ensure the accuracy of the result in this study, we excluded elevation, and then included all the remaining variables in the MGWR model. The bi-square kernel function with adaptive distance and corrected Akaike information criterion (AIC) was chosen as the parameters of the MGWR model. Table 4 shows that *R*^2^ and *R*^2^*adj* of the model with OPD as the dependent variable were 0.917 and 0.880, respectively, and *R*^2^ and *R*^2^*adj* of the model with OPP as the dependent variable were 0.849 and 0.770, respectively. The model fit for both was great, while the accuracy of the model with OPD was significantly better than that of the model with OPP, implying that the factors selected in this study have more substantial explanatory power for the OPD and relatively weaker explanatory power for the OPP.

**Table 4:** Comparison of model results.

**Dependent variable**	**AIC**	**RSS**	***R^2^***	***R^2^adj***
OPD	85.990	7.196	0.917	0.880
OPP	143.555	13.121	0.849	0.770

AIC indicates Akaike Information Criterion, RSS indicates Residual Sum of Squares, *R*^2^ indicates Coefficient of Determination, *R*^2^adj indicates Adjusted Coefficient of Determination.

#### 3.3.3 Analysis of influencing factors

**Figure 5:**
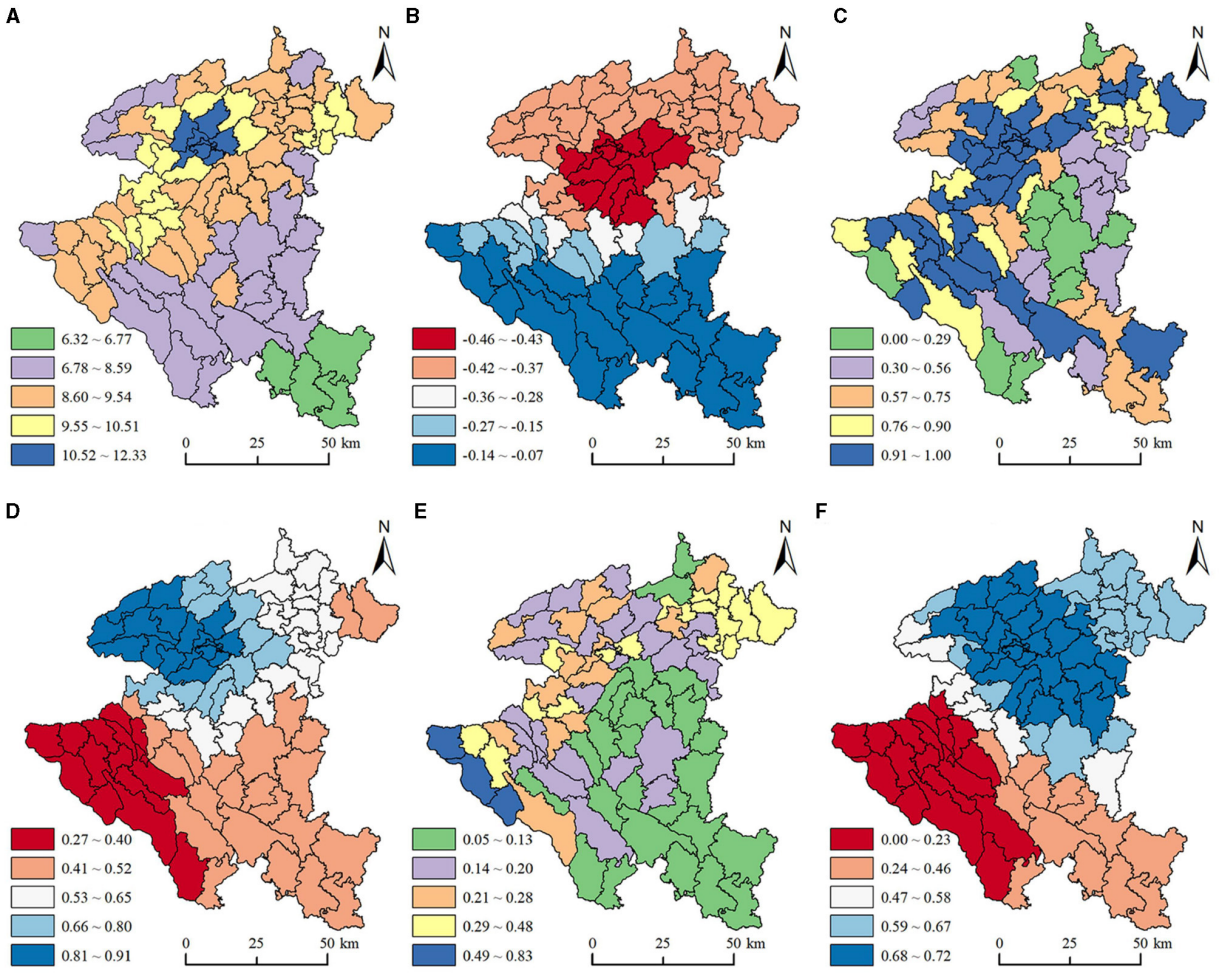
Spatial distribution of the factors and regression coefficients. **(A)** Air quality. **(B)** Coefficient of air quality on OPD. **(C)** Karst proportion. **(D)** Coefficient of karst proportion on OPP. **(E)** Rocky desertification. **(F)** Coefficient of rocky desertification.

In this study, the sign and value of the mean coefficient were used to represent the direction and intensity of the influencing factors. Tables 5, 6 show that significant differences existed in different factors, and even the same factors displayed intensity differences in different townships, with obvious spatial heterogeneity. To specifically reveal the mechanism of influencing factors, we plotted the distribution of regression coefficients for three typicality factors, including air quality, karst proportion, and rocky desertification (Figure 5).

**Table 5:** The estimation results of the MGWR model on OPD.

**Variables**	**Mean**	**STD**	**Min**	**Median**	**Max**	***p* ≤ 5 (%)**
Intercept	0.155	0.010	0.134	0.156	0.169	100.00
Slope	−0.016	0.015	−0.049	−0.014	0.005	0.00
Rainfall	0.103	0.016	0.080	0.104	0.129	0.00
Temperature	−0.024	0.009	−0.038	−0.025	−0.010	0.00
Air quality	0.307	0.143	−0.455	−0.398	−0.075	66.67
River density	−0.068	0.021	−0.095	−0.073	−0.023	0.00
GDP per capita	0.054	0.003	0.047	0.054	0.059	0.00
CLC	0.673	0.003	0.669	0.672	0.681	100
CLA	−0.153	0.010	−0.169	−0.155	−0.135	0.00
URS	0.011	0.007	−0.001	0.011	0.025	0.00
Ethnic structure	0.170	0.004	0.162	0.170	0.178	100
Medical level	−0.187	0.006	−0.196	−0.187	−0.171	100
Education level	−0.055	0.012	−0.073	−0.056	−0.036	0.00
Migration rate	−0.007	0.028	−0.060	−0.006	0.041	0.00
Karst proportion	0.004	0.010	−0.018	0.008	0.015	0.00
KLI	0.105	0.016	0.072	0.114	0.123	0.00
KLA	0.012	0.015	−0.012	0.012	0.039	0.00
Rocky desertification	0.502	0.243	−0.003	0.631	0.723	85.06

STD indicates Standard Deviation.

**Table 6:** The estimation results of the MGWR model on OPP.

**Indicates**	**Mean**	**STD**	**Min**	**Median**	**Max**	***p* ≤ 5 (%)**
Intercept	0.014	0.030	−0.041	0.015	0.064	0.00
Slope	−0.413	0.007	−0.432	−0.411	−0.404	0.00
Rainfall	−0.015	0.015	−0.057	−0.011	0.012	0.00
Temperature	−0.136	0.008	−0.155	−0.137	−0.121	0.00
Air quality	−0.398	0.042	−0.513	−0.392	−0.306	100.00
River density	0.130	0.026	0.083	0.126	0.181	1.15
GDP per capita	−0.037	0.129	−0.217	−0.046	0.308	0.00
CLC	0.512	0.002	0.508	0.512	0.518	100.00
CLA	−0.051	0.105	−0.189	−0.062	0.158	0.00
URS	−0.174	0.019	−0.212	−0.173	−0.140	0.00
Ethnic structure	−0.201	0.011	−0.219	−0.203	−0.176	81.61
Medical level	−0.144	0.003	−0.153	−0.144	−0.139	0.00
Education level	−0.123	0.028	−0.179	−0.108	−0.090	0.00
Migration rate	−0.782	0.009	−0.796	−0.784	−0.763	100.00
Karst proportion	0.601	0.189	0.272	0.553	0.911	58.62
KLI	−0.357	0.013	−0.389	−0.353	−0.334	0.00
KLA	−0.074	0.080	−0.229	−0.068	0.038	0.00
Rocky desertification	−0.168	0.021	−0.209	−0.165	−0.129	5.75

STD indicates Standard Deviation.

##### 3.3.3.1 Natural factors

Slope and temperature displayed a weak negative correlation with OPD (Table 5) and a strong negative correlation with OPP (Table 6). Due to the small difference in slope and temperature at the township scale, the climate and topographic conditions of different townships were relatively close, and this similarity led to the relatively weak influence of slope and temperature changes on regional OPD. In addition, due to the many restrictions on the activities of the older adult population and their sensitivity to temperature, they often chose to live in a place with a gentle slope and suitable temperature. Such residence preference and migration avoidance behavior resulted in the relatively strong influence of slope and temperature on OPP. We also found a strong positive correlation between rainfall and OPP (Table 6) and a weak correlation between rainfall and OPD (Table 5). To a certain extent, rainfall can bring a good ecological environment and suitable climatic conditions, which continuously attracts the older adult population to migrate or live, and has a certain impact on regional OPP and OPD. Xu et al. (39) established that areas with abundant rainfall have a “pull” effect on the older adult population. We found that there was a positive correlation between river density and OPP (Table 6). One possible explanation is that townships with higher river density have greater natural beauty and green levels. The environmental features provide the older adult population with opportunities for leisure, walking, and enjoying nature, extending their life expectancy to a certain extent (5) and leading to an increase in the proportion of older adults.

What is interesting is that the air quality in 66.67% of the townships showed a strong positive correlation with OPD at p = 0.05 level (Table 5), while the air quality of all townships showed a significant negative correlation with OPP at p = 0.05 level (Table 6). In general, the higher the concentration of PM2.5, the worse the environmental quality, seriously threatening the life, safety, and health of older adults (5). Our study found that the air quality in Anshun gradually deteriorated from south to north (Figure 5A), and the absolute value of the coefficient of air quality on OPD presented a spatial feature of “small in the south, large in the north, and convex in the middle” (Figure 5B). The main reason is that there are more industrial enterprises in the central and northern parts of Anshun, and the impact of air quality on OPD in the northern parts of Anshun was greater than that in the southern parts. However, it is worth noting that the impact of air quality on the OPD is not referential, and residential inertia, location characteristics, medical security conditions, and other socioeconomic factors also affect OPD.

##### 3.3.3.2 Economic factors

There was no significant correlation between GDP per capita and OPD and OPP (Tables 5, 6), which suggests that the spatial distribution of economic levels does not coincide with the spatial distribution of population aging (22). A possible explanation for this may be due to the combined effects of resource allocation, policy orientation, population flow, industrial structure, and other factors; adjacent towns and villages also show certain similarities and synergies in the process of economic development. The spatial heterogeneity of the economic level represented by the per capita GDP of the township scale is small (16); therefore, the impact on OPD and OPP is not significant. The marginal effect of economic level on life expectancy also reduced the reliance on the economy for population aging (40). The coefficient of land for construction (CLC) of all townships displayed a significant positive correlation with OPD and OPP at p = 0.05 level (Tables 5, 6). This indicates that CLC has an important effect on the proportion and density of the older adult population in Anshun City. Construction land is the carrier of urban development and population agglomeration, which reflects the development intensity and scale of land resources (41). As the availability of land resources in karst areas is limited, townships with high CLC have higher development levels and more complete infrastructure. Therefore, more attention should be paid to the relationship between Anshun's aging population and the development scale of local construction land. We found a negative correlation between the coefficient of arable land (CLA) and OPD (Table 5) and a weak negative correlation between CLA and OPP (Table 6). The industrial structure of townships with higher CLA is dominated by agriculture and the capacity to accommodate older adults is less than in the industrial town. The urban and rural structure showed a negative correlation with OPP (Table 6). Townships with high administrative levels have a larger job market and are highly attractive to young people. Consequently, the age structure of the population in these townships is younger than in townships with low administrative levels (42), which then affects the regional OPP-specific gravity.

##### 3.3.3.3 Social factors

All the ethnic structures of the township displayed a significant positive correlation with OPD at p = 0.05 level (Table 5), while the ethnic structure in 81.61% of the townships showed a significant negative correlation with OPP at p = 0.05 level (Table 6). Generally speaking, the socioeconomic development of ethnic townships is underdeveloped, the construction of infrastructure is relatively imperfect, the development of social undertakings is relatively lagging behind, and the level of facilities and services in education, health, culture, and other aspects needs to be improved. This results in a relatively low absolute number of older adult population in these townships. The poor natural environment and fragile ecological environment in the region combined with the sluggish socioeconomic development further cause the outflow of young people, resulting in a relatively high proportion of older adults (38, 43).

In this study, a strong negative correlation was found between the level of medical facilities and OPP (Table 6) which differs from previous studies. This result may be related to the influence the level of medical facilities have on population aging. The level of medical facilities, through life expectancy and fertility rate, affect OPP (44). Townships with abundant medical resources attract a young migrant population, and the arrival of migrant populations means that a relatively young age structure is introduced into these areas. To some extent, this offsets the increase in average life expectancy of urban and rural residents brought about by the improvement of medical resources, resulting in a decline in the proportion of older adult population.

Education level displayed a negative correlation with OPP (Table 6) and a weak correlation with OPD (Table 5). In recent years, the investment in education in China has led to a gradual increase in the average educational attainment of the population. However, the average years of education in Anshun is only 8.26 years (compared to the average years of schooling for the population aged 15 and above in China in 2020 was 9.91 years), indicating that this region lags behind in education development. In general, more educated areas tend to have more opportunities and resources to attract young people to work and live, making the age structure more youthful. The migration rate across all townships showed a significant negative correlation with OPP at p = 0.05 level (Table 6). The migration rate had a dominant effect on OPP at the township scale. The age structure of the migrant population was dominated by young people. The economically backward townships showed significant characteristics of out-migration, which accelerated the aging structure of the local population (5).

##### 3.3.3.4 Karst factors

A strong positive correlation was found between karst proportion and OPP in this study (Table 6). Due to karst areas having special geographical environments and geological conditions (31), a higher proportion of karst areas are faced with problems such as poor soil and fragile ecological environment. This compels the young population to seek out greater possibilities and abilities to find better survival and development opportunities in the face of adverse environmental conditions. To a certain extent, the population pressure and environmental burden in these townships can be alleviated, which makes the proportion of OPP increase. It can be seen from Figure 5C that the northwest townships and southwest townships have the largest karst proportion. However, in Figure 5D the coefficients of karst proportion on OPP reveal a spatial pattern of high in the northwest and low in the southwest. The reason is that the young population in the towns and villages with high karst proportion in the northwest of Anshun is easily affected by the siphoning effect from Guiyang, the neighboring provincial capital city, and chooses to enter the city for employment, which makes the OPP in these places increase. In the southwest towns and villages with low karst proportion, the older adult population often chooses to go to cities with better economic and social development in order to benefit from better medical security conditions and perfect social service infrastructure, resulting in a decrease in the OPP.

Karst lithology showed a strong positive correlation with OPD (Table 5) and a strong negative correlation with OPP (Table 6). Dolomite, used to characterize lithology, has greater lithology hardness. Because of its easy availability and people's production and living needs, dolomite becomes the first choice for house construction, thus resulting in relatively large urban settlements in areas with more demand (45). Additionally, population aging shows a noticeable trend of urban and rural inversion, which means the OPP in urban is lower than in rural areas (38). Therefore, townships with dolomite as the dominant lithology have higher OPD and lower OPP. We found no significant correlation between karst landform and OPD and OPP (Tables 5, 6), indicating that the distribution of the older adult population had no specific preference in landform type. This phenomenon may be significantly associated with the smaller topographic relief of Anshun (46).

Rocky desertification in 81.61% of the townships showed a significant positive correlation with OPD at *p* = 0.05 level (Table 5), while it showed a strong negative correlation with OPP (Table 6). It can be seen from Figure 5E that the townships with deeper rocky desertification are mainly located in the north and west of Anshun. The coefficient of rocky desertification on OPD is most significant in the economically developed regions represented by Xixiu District and Puding County, but the southwest has the lowest influence coefficient (Figure 5F). Previous studies have found that the increase in population and affluence will lead to more serious rocky desertification (47). Although Xixiu District and Puding County in the northwest are also in the serious rocky desertification area, as the central cities of Anshun, they are driven by the radiation of Guiyang, the provincial capital of the Guizhou province. Their economic and social development and medical services are relatively good, prompting the older adult population from other areas with a high degree of rocky desertification to migrate to live. However, although the areas with high rocky desertification degree in the southern region are faced with a fragile ecological environment, relatively backward economic and social development, and imperfect basic service facilities for older adults, the older population continues to live in the same place due to factors such as long-term living habits, family and social network. Older adults appear relatively less responsive to the rocky desertification. Thus, the influence on the change of regional OPD is relatively small. But at the same time, it should be noted that factors such as the outflow of young people in the serious rocky desertification area have increased the local OPP, and the phenomenon of the intensification of population aging also needs further attention.

## 4 Discussion

### 4.1 Research implications

The spatial distribution of population aging in karst areas has obvious regional differences. Karst areas are an essential part of the complicated terrain region in southwest China, having their own unique geo-geological features that further influence the process of population aging and its spatial pattern in these areas, resuting in obvious regional differences. It is generally believed that suburbanization and reverse urbanization are the results of the late stage of urban development. Further, the social resources such as pension and medical are more evenly distributed in developed cities under the township scale. However, the OPP of Anshun shows a spatial pattern of “small concentration and large scattering,” and a large number of the older adult population is concentrated in townships with small areas, which leads to the increasingly obvious Matthew effect of older people population distribution. There are significant differences in the influence mechanism of economic development level on the spatial distribution of population aging at different scales. Economic development level is the influencing factor of the spatial distribution of population aging at the county or larger scale (23), but its contribution to spatial distribution of population aging at the township scale is not outstanding (16). The spatial heterogeneity of the economic development level at the township scale is smaller than at the county or larger scale, which suggests the effect of economic development level on the spatial distribution of population aging is constrained by spatial heterogeneity and the size of the study area.

However, the economic mechanism of population aging research is more urgent and complex. Whether population aging affects economic growth or economic growth affects population aging is a topic worth thinking about in the process of population aging research. Some scholars point out that population aging affects labor productivity, but this impact is not linear, and it is influenced by a variety of factors. Meanwhile, the social mechanism of population aging research is also worthy of attention. In general, the impact of social factors on population aging contains both direct and indirect pathways, and the negative impact formed by the indirect pathway is much greater than the positive impact of the direct pathway (10). This supposition is also consistent with the view of Chang and Ao (44).

### 4.2 Policy suggestion

China has a refined traditional culture of respect for older adults from ancient times and also has the institutional advantages necessary to deal with the effects of aging. The population aging problem has brought forth new opportunities for socioeconomic development, such as the expansion of the older adult consumer market and the improvement of labor quality. Simultaneously it has also brought many challenges such as increasing pressure on social security and rising demand for medical and health services. Anshun City's older adult population has grown both in number and percentage in recent years, and the rate of aging is getting acute. In order to effectively handle these problems, a number of important steps still need to be aggressively pursued.

In the future, Anshun should reduce the decreasing trend in the natural population growth rate, actively implement measures related to parenthood, education, childcare, and other sectors, and hasten the development of a system to support fertility policies. It should continue to improve endowment insurance, medical insurance, and other social security systems, as well as the creation of social security systems for older adults in towns and villages, to guarantee that they have access to basic healthcare and life protection. To address the labor shortage brought on by an aging population, towns and villages should be encouraged to build industries that fit their unique qualities, and young people should be encouraged to establish businesses and find employment in their hometowns. To raise the skill level and employability of older adults, the government must intensify their training and guidance. It should enhance township health and medical services, fortify the development of village clinics and township health facilities, and raise the standard of healthcare provided at the community level. The government should also keep pushing for the growth and prosperity of older adult care services in cities and rural areas, aggressively encourage social forces to get involved in older adult care services, support the integration of older adult care services with other services such as medical attention and rehabilitation, and cater to the various service needs of older adults. The state should also keep enhancing the services provided for older adults in towns and communities, organize cultural events and sports that are appropriate for them, and enrich their spiritual and cultural life.

### 4.3 Research shortcomings and prospects

The present study only discusses the differences and influencing factors of the spatial distribution of population aging in a single cross-section time. Further data collection is needed in the future to carry out long-term behavior research on population aging. Big data and traditional data should be actively combined in population aging research going forward to compensate for their individual shortcomings in sample size, coverage, and quality. Data mining and machine learning technologies should be used to do in-depth analysis of massive data, including social media, electronic health records, and mobile applications. To learn more about the regional population aging trend, spatial pattern, and affecting factors, more precise and comprehensive data should be utilized. At the same time, township birth rate, mortality rate, immigration rate, and other data may be more important in the study of the mechanism of population aging at the township and other fine scales, but given the difficulty in data acquisition, more detailed data need to be collected in the future to supplement this part of the study. Whether the study of the influencing mechanism on the spatial pattern of population aging in Anshun City at the township scale is universal needs to be tested by further using other regions as examples. Driven by the “flow space” (such as population flow, information flow, material flow, etc.), the core issue of population aging research will be transformed into how to understand and respond to the aging challenges and opportunities brought by population flow. In the future, complexity science should be applied to further explore this problem.

## 5 Conclusion

(1) Anshun City's OPP is generally high; more than 7% of the population in Anshun are older adults, making it part of an aging society. The OPP showed a trend of increasing gradually from west to east and appeared “high in the east and low in the west” in spatial distribution. The OPD presents a trend of increasing gradually from south to north in space. The distribution of the older adult population in the vast majority of townships is relatively scattered. Still, a few townships were oversaturated with older adult populations, showing the characteristics of “small concentration and large scattering.”

(2) At the township scale, both the OPP and the OPD showed significant spatial positive correlation, and the spatial agglomeration characteristics are obvious. The OPD and OPP have a positive spatial correlation in global scope, and townships with similar OPP or OPD are spatially adjacent. In the OPD local spatial agglomeration type, high-high types of townships were mainly distributed in the central parts of the Xixiu District, and low-low types of townships were distributed in the southern parts of Anshun in the form of clusters. In the OPP local spatial agglomeration type, high-high types of townships were concentrated in the northeast boundary of Anshun, and low-low types of townships were concentrated in Songqi town, Yaopu town, and Baiyan town, showing a coupling relationship with townships with high-economic levels.

(3) The spatial distribution characteristics of population aging were the consequence of complex contributions such as natural factors, social factors, economic factors, and karst factors. The coefficient of land for construction, and migration rate played a dominant role in OPD and OPP, respectively. Air quality, ethnic structure, karst lithology, and rocky desertification displayed a strong influence on both OPD and OPP. Rainfall, river density, medical level, and coefficient of arable land had a certain effect on OPD. Slope, urban and rural structure, and karst proportion had only a modest effect on OPP. The spatial distribution characteristics of population aging were not influenced by karst landform, GDP per capita, temperature, or educational attainment.

## Data availability statement

The original contributions presented in the study are included in the article/supplementary material, further inquiries can be directed to the corresponding author.

## Author contributions

XZ: Writing—review & editing, Writing—original draft, Visualization, Software, Project administration, Methodology, Data curation, Conceptualization. JS: Writing—review & editing, Writing—original draft, Software, Data curation. MC: Writing—original draft, Software, Methodology, Data curation. JY: Writing—review & editing, Writing—original draft, Supervision, Methodology, Data curation, Conceptualization.

## References

[1] Yenilmez MI. Economic social consequences of population aging the dilemmas and opportunities in the Twenty-First Century. Appl Res Qual Life. (2015) 10:735–52. 10.1007/s11482-014-9334-2

[2] Man W Wang SB Yang H. Exploring the spatial-temporal distribution and evolution of population aging and social-economic indicators in China. BMC Public Health. (2021) 21:1–13. 10.1186/s12889-021-11032-z 34020620 PMC8140474

[3] Lu JH Liu Q. The characteristics, influence and coping strategies of the new form of aging society in China: interpretation based on the data of the seventh census. Populat Econ. (2021) 42:13–24. 10.3969/j.issn.1000-4149.2021.00.036 (in Chinese).

[4] Wu FF Yang HN Gao B Gu Y. Old, not yet rich? the impact of population aging on export upgrading in developing countries. China Econ Rev. (2021) 70:101707. 10.1016/j.chieco.2021.101707

[5] Wu YY Song YX Yu TT. Spatial differences in China's population aging and influencing factors: the perspectives of spatial dependence and spatial heterogeneity. Sustainability. (2019) 11:5959. 10.3390/su11215959

[6] Barak Y Neehoffs S Glue P. Ageing badly: indicators of old-age structure in Australia and New Zealand. J Prim Health Care. (2020) 12:272–6. 10.1071/HC19095 32988449

[7] Xu Z Lin XS Luo CY. Temporal spatial evolution and classification of population aging in Chongqing. J. Beijing Norm. Univer. (2019) 55:772–9. 10.16360/j.cnki.jbnuns.2019.06.014 (in Chinese).

[8] Reynaud C Miccoli S Lagona F. Population ageing in italy: an empirical analysis of change in the ageing index across space and time. Spatial Demography. (2018) 6:235–51. 10.1007/s40980-018-0043-6

[9] Zhou R Zhuang RL Huang CX. Pattern evolution and formative mechanism of aging in China. Acta Geographica Sinica. (2019) 74:2163–77. 10.11821/dlxb201910015 (in Chinese).

[10] Kashnitsky I Beer JD Wissen LV. Unequally ageing regions of Europe: exploring the role of urbanization. Populat Stud. (2021) 75:221–37. 10.1080/00324728.2020.1788130 32700651

[11] Chen Y Bouferguene A Shen YH Al-Hussein M. Difference analysis of regional population ageing from temporal and spatial perspectives: a case study in China. Reg Stud. (2019) 53:849–60. 10.1080/00343404.2018.1492110

[12] Robert SY. The standard deviational ellipse; an updated tool for spatial description. Geografiska AnnalerSeries B, Human Geogr. (1971) 53:28–39. 10.1080/04353684.1971.11879353

[13] Wang SB. Spatial patterns and social-economic influential factors of population aging: a global assessment from 1990 to (2010). Soc Sci Med. (2020) 253:112963. 10.1016/j.socscimed.2020.112963 32289647

[14] Guan DY Lei L Han ZL. Spatial-temporal variation of population aging: a case study of China's Liaoning Province. Complexity. (2020) 2020:1–13. 10.1155/2020/5436061

[15] Zhao DX Han ZL Wang L. The spatial pattern of aging population distribution and its generating mechanism in China. Acta Geographica Sinica. (2017) 72:1762–75. 10.11821/dlxb201710003 (in Chinese).

[16] Li SX Wang XZ Ji XL Zhang Y. Spatial change and influencing factors of population aging in Shandong Province at the township scale. Progr Geog. (2019) 38:567–76. 10.18306/dlkxjz.2019.04.009

[17] Zhou SH Xie M Kwan MP. Ageing in place and ageing with migration in the transitional context of urban China: a case study of ageing communities in Guangzhou. Habitat Int. (2015) 49:177–86. 10.1016/j.habitatint.2015.05.022

[18] de Schrijver E Bundo M Ragettli MS Sera F Gasparrini A Franco OH. Nationwide analysis of the heat- and cold-related mortality trends in Switzerland between 1969 and 2017: the role of population aging. Environ Health Perspect. (2022) 130:037001. 10.1289/EHP9835 35262415 PMC8906252

[19] Ying K Li XD Cheng DY. Spatial pattern evolution and environmental causes of population aging in Guizhou Province. Res Environm Yangtze Basin. (2020) 29:334–45.

[20] Gu D Zhang Z Zeng Y. Access to healthcare services makes a difference in healthy longevity among older people Chinese adults. Soc Sci Med. (2009) 68:210–9. 10.1016/j.socscimed.2008.10.025 19038485 PMC3693546

[21] Rogerson PA. The geography of older people minority populations in the United States. Int J Geograph Inform Sci. (1998) 12:687–98. 10.1080/136588198241608 12348934

[22] Wang S Luo K Liu Y Zhang S Lin X Ni R. Economic level and human longevity: spatial and temporal variations and correlation analysis of per capita GDP and longevity indicators in China. Arch Gerontol Geriatr. (2015) 61:93–102. 10.1016/j.archger.2015.03.004 25847813

[23] Wu LX Huang ZY Pan ZH. The spatiality and driving forces of population ageing in China. PLoS ONE. (2021) 16:e243559. 10.1371/journal.pone.0243559 33428682 PMC7799793

[24] Han XL Li JM Wang NN. Spatiotemporal evolution of Chinese ageing from 1992 to 2015 based on an improved Bayesian space-time model. BMC Public Health. (2018) 18:1–10. 10.1186/s12889-018-5417-6 29661176 PMC5902982

[25] Li J Han X Zhang X Wang S. Spatiotemporal evolution of global population ageing from 1960 to (2017). BMC Public Health. (2019) 19:1–15. 10.1186/s12889-019-6465-2 30700286 PMC6354390

[26] Lewandowska-Gwarda K Antczak E. Urban ageing in Europe—spatiotemporal analysis of determinants. Int J Geo-Inform. (2020) 9:413. 10.3390/ijgi9070413

[27] Cheng Y Gao S Li S Rosenberg M. Understanding the spatial disparities and vulnerability of population aging in China. Asia Pacific Policy Stud. (2019) 6:73–89. 10.1002/app5.267

[28] Chen W Bai S Zhao H Han X Li L. Spatiotemporal analysis and potential impact factors of vegetation variation in the karst region of Southwest China. Environ Sci Pollut Res. (2021) 28:61258–73. 10.1007/s11356-021-14988-y 34170472

[29] Cheng WM Zhou CH Chai HX Liu H Zhou Z. Research and compilation of the geomorphologic atlas of the People's Republic of China (1:1,000,000). J Geograph Sci. (2011) 21:89–100. 10.1007/s11442-011-0831-z

[30] Goodman AC. Using Lorenz curves to characterise urban older people populations. Urban Stud. (1987) 24:77–80. 10.1080/00420988720080071

[31] Shi SN Xie BG Hu BQ Tang C Yan Y Li X. The relationship between population distribution characteristics and natural factors in the karst mountainous area of the Northwestern Guangxi, China. Scientia Geographica Sinica. (2019) 39:1484–95. 10.13249/j.cnki.sgs.2019.09.014 (in Chinese).

[32] Deng W Cheng YF Yu H Peng L Kong B Hou Y. Spatio-temporal characteristics of population and economy in transitional geographic space at the southern end of “Hu Huan-yong Line”. J Mountain Sci. (2022) 19:350–64. 10.1007/s11629-021-6846-8

[33] Yu HC Fotheringham AS Li ZQ Osham T Kang W Wolf J. Inference in multiscale geographically weighted regression. Geograph Analy. (2020) 52:87–106. 10.1111/gean.12189

[34] Fotheringham AS Yang WB Kang W. Multiscale geographically weighted regression (MGWR). Ann Am Assoc Geograph. (2017) 107:1247–65. 10.1080/24694452.2017.1352480

[35] Oshan TM Li ZQ Kang W Wolf LJ Fotheringham S. MGWR: a python implementation of multiscale geographically weighted regression for investigating process spatial heterogeneity and scale. In J Geo-Inform. (2019) 8:269. 10.3390/ijgi8060269

[36] Li ZQ Fotheringham AS Oshan TM Wolf LJ. Measuring bandwidth uncertainty in multiscale geographically weighted regression using Akaike weights. Ann Am Assoc Geograph. (2020) 110:1500–20. 10.1080/24694452.2019.1704680

[37] Xu X Zhao Y Guo AM. Spatial distribution of the older people population in Nanjing based on the street scale. Human Geography. (2016) 31:88–94. 10.13959/j.issn.1003-2398.2016.06.011 (in Chinese).

[38] Ebeling M Rau R Sander N Kibele E Klüsener S. Urban–rural disparities in old-age mortality vary systematically with age: evidence from Germany and England & Wales. Public Health. (2022) 205:102–9. 10.1016/j.puhe.2022.01.023 35276525

[39] Xu X Zhao Y Zhang XL. Identifying the impacts of social, economic, and environmental factors on population aging in the yangtze river delta using the geographical detector technique. Sustainability. (2018) 10:1528. 10.3390/su10051528

[40] Zhang K Sun H Li XY. Aging population spatial distribution discrepancy and impacting factor. Sustainability. (2022) 14:9528. 10.3390/su14159528

[41] Luo J Shi PJ Zhang XB. Population distribution and influencing factors in Lanzhou-Xi'ning urban agglomeration based on township scale. J Arid Land Res Environm. (2020) 34:104–111. 10.13448/j.cnki.jalre.2020.190 (in Chinese).

[42] Smailes P Griffin T Argent N. Demographic change, differential ageing, and public policy in rural and regional Australia: a three-state case study. Geographical Res. (2014) 52:229–49. 10.1111/1745-5871.12067

[43] Dou X Liu Y. older people migration in China: types, patterns, and determinants. J Appl Gerontol. (2017) 36:751–71. 10.1177/0733464815587966 26081931

[44] Chang L Ao RJ. Spatio-temporal pattern and influencing mechanism analysis of population ageing in China's border counties. World Regional Stud. (2021) 30:410–421. 10.3969/j.issn.1004-9479.2021.02.2019444 (in Chinese).

[45] Li XQ Yang QN Zhou KC Luo W. Distribution characteristics of rural settlement on different lithology in karst area: a case study of Pingguo City. Carsologica Sinica. (2021) 40:355–62. 10.11932/karst20210212 (in Chinese).

[46] Li XD. The Influence of Space Structure of Population In Karst Plateau and Mountain Area on Sustainable Development: as the Case of Guizhou Province. Shanghai: East China Normal University. (2007).

[47] Wu XQ Liu HM Huang XL Zhao T. Human driving forces: analysis of rocky desertification in karst region in Guanling County, Guizhou Province. Chin Geographical Sci. (2011) 21:600–8. 10.1007/s11769-011-0496-7

